# Heterogeneity of odorant identification impairment in patients with Alzheimer’s Disease

**DOI:** 10.1038/s41598-017-05201-7

**Published:** 2017-07-06

**Authors:** Yumi Umeda-Kameyama, Shinya Ishii, Masashi Kameyama, Kenji Kondo, Atsushi Ochi, Tatsuya Yamasoba, Sumito Ogawa, Masahiro Akishita

**Affiliations:** 1 Department of Geriatric Medicine, Graduate School of Medicine, The University of Tokyo, Tokyo, 113-8655 Japan; 2 Department of Diagnostic Radiology, Tokyo Metropolitan Geriatric Hospital and Institute of Gerontology, Tokyo, 173-0015 Japan; 3 Department of Otolaryngology, Graduate School of Medicine, The University of Tokyo, Tokyo, 113-8655 Japan

## Abstract

Alzheimer’s disease (AD) patients exhibit olfactory dysfunction. However, the olfactory declineti precise nature is not fully understood. One hundred patients (60 AD, 28 amnestic mild cognitive impairment (aMCI), 12 Normal) were enrolled. All participants underwent olfactory function testing using an odour stick identification test for Japanese (OSIT-J). OSIT-J scores were significantly correlated with recall. We classified OSIT-J odorants into three groups: Category I, odorants that were difficult for normal aged subjects to identify; Category II, odorants that became harder to accurately identify with cognitive decline; and Category III, odorants that even AD patients could identify. We defined a “*cognitive subset*” consisting of six Category II OSIT-J odorants (perfume, rose, Japanese cypress, curry, India ink and gas leak odour). The ability to identify “*cognitive subset*” odours was significantly better indicator of cognitive status than the ability to identify “non-cognitive subset”, which consisted of the six remaining items. The ability to identify the gas leak odorant was decreased early in the aMCI stage, suggesting a need to reconsider the odours used to signal gas leaks. The “*cognitive subset*” would provide a more convenient and effective biomarker for diagnosing dementia in clinical settings.

## Introduction

Alzheimer’s disease (AD) has long been reported to be associated with olfactory dysfunction^1–4^. AD-associated olfactory dysfunction is related to recall function decline^5–8^. However, the relationship between olfaction and specific neural substrates of cognitive function has not been well explored. This relationship may be attributable to the neurofibrillary tangle in the entorhinal cortex and hippocampus^9^ and cholinergic system dysfunction^10^.

We strongly believe that olfactory testing is a clinically useful non-invasive tool for evaluating disease state in elderly people. It provides much information about dementia and can predict progression from amnestic mild cognitive impairment (aMCI) to AD^11^, but little is known about the precise nature of AD-associated olfactory decline. Most contributions have studied total scores on olfactory examinations, but it is unknown whether olfactory decline occurs equally for all odours. If the inability to identify a specific odour indicates susceptibility to AD, then that odor may serve as a “canary in a coal mine” to detect AD pathology.

Olfactory testing is laborious. Stick tests such as the Odour Stick Identification Test for Japanese (OSIT-J)^12^ are certainly more convenient than conventional testing with an olfactometer, but they are still burdensome in a typical clinical setting. To reduce the burden, an OSIT-J subset indicating AD susceptibility may provide a convenient diagnostic test for dementia. Furthermore, we wish to gain some clues to improving meal enjoyment for AD patients.

To investigate the heterogeneity of AD-associated olfactory decline with these objectives, we performed olfactory tests and cognitive examinations with AD patients.

## Results

### Demographics

Our participants’ demographic characteristics are detailed in Table 1. ANOVA comparisons of the Normal, aMCI and AD groups found no significant differences in age, Barthel Index, instrumental activities of daily life (IADL) or 15-item Geriatric Depression Scale scores (GDS15). *χ* square analysis revealed no significant intergroup differences in sex, hypertension, hyperlipidaemia or diabetes. ANOVA did, however, reveal significant difference in Mini-Mental State Examination (MMSE) and OSIT-J scores (*p* < 0.001).

**Table 1:** Participants characteristics.

	Normal	aMCI	AD
*n* (male/female)	12 (1/11)	28 (10/18)	60 (19/41)
age (year)	77.1 ± 6.4	81.0 ± 6.0	80.5 ± 6.5
age range	65–87	70–93	62–92
Barthel Index	96.3 ± 5.3	95.8 ± 6.0	93.5 ± 12.6
IADL	6.7 ± 2.0	5.8 ± 2.1	5.2 ± 2.2
MMSE	26.6 ± 3.9	24.7 ± 2.7	21.0 ± 3.3***
GDS15	4.2 ± 2.4	5.6 ± 3.6	5.8 ± 3.5
Hypertension	2 (15%)	12 (44%)	24 (40%)
Hyperlipidaemia	4 (31%)	9 (33%)	17 (28%)
Diabates	2 (15%)	4 (15%)	13 (22%)
OSIT-J total	7.3 ± 2.4	5.0 ± 3.1*	3.6 ± 2.4***

* and *** denote significant (*p* < 0.05 and *p* < 0.001, respectively) differences from normal subjects by Tukey-Kramer honest significant difference test. Numerals of age, Barthel index, MMSE, GDS15 and OSIT-J are averages ± standard deviations and that of hypertention, hyperlipidaemia and diabetes are *n* (%). Abbreviations: aMCI, amnestic mild cognitive impairment; AD, Alzheimer’s disease; IADL, instrumental activity of daily living; MMSE, Mini-Mental State Examination; GDS15, 15-item Geriatric Depression Scale; OSIT-J, odour stick identification test for Japanese.

OSIT-J scores of 8 are usually considered normal, but OSIT-J scores decline with age. A score of six, indicating an identification accuracy of approximately 50%, would be considered normal for octogenarians.

**Figure 1:**
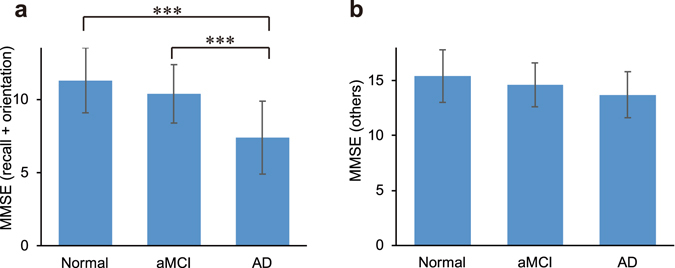
MMSE subsets and cognitive status. (**a**) MMSE recall and orientation 13-item subset for each cognitive status. (**b**) MMSE subset of 17 other items for each cognitive status. Error bars are standard deviations. ***denotes significant (*p* < 0.001) difference with Bonferroni correction. Abbreviations: MMSE, Mini-Mental State Examination.

Both the recall and orientation MMSE subset and the remaining items MMSE subset showed decreases correlating with the development of cognitive impairment. However, the recall and orientation subset showed a steeper decline that was statistically significant (Fig. 1).

### Cognitive function and olfactory function

OSIT-J scores were significantly correlated with total MMSE scores, after adjusting for age, sex, hypertension and diabetes. OSIT-J scores were significantly correlated (*p* < 0.001) with recall and orientation MMSE subset scores (13 items) after adjusting for age, sex, hypertension and diabetes. However, they were not significantly correlated (*p* = 0.55) with the remaining 17 MMSE items. Recall showed a much higher standardized coefficient (*β*) than temporal and spacial orientation in multiple regression analysis with covariates of age, sex, hypertension and diabetes (Table 2). The standardized coefficient of recall alone almost equalled that of recall and orientation, showing that the contribution of orientation was minimal. Among the four covariates (age, sex, hypertension, diabetes), only age consistently significantly associated with OSIT-J scores.

**Table 2:** Adjusted associations of MMSE and OSIT-J scores.

Variates	*β*	%V	*p*	*R* ^2^
Recall + orientation(13)	0.41	18.4	<0.001	0.27
Other scores (17)	0.06	2.9	0.55	0.12
Recall (3)Orientation (10)	0.410.31	18.39.9	<0.0010.004	0.270.20

*β* and %V denote standardized coefficient and %variance explained, respectively. The numbers in parentheses denote allotment of Mini-Mental State Examinations (MMSE) items. All three multiple regression analysis models included age, sex, hypertension and diabetes as covariates.

### Odorant differences

**Figure 2:**
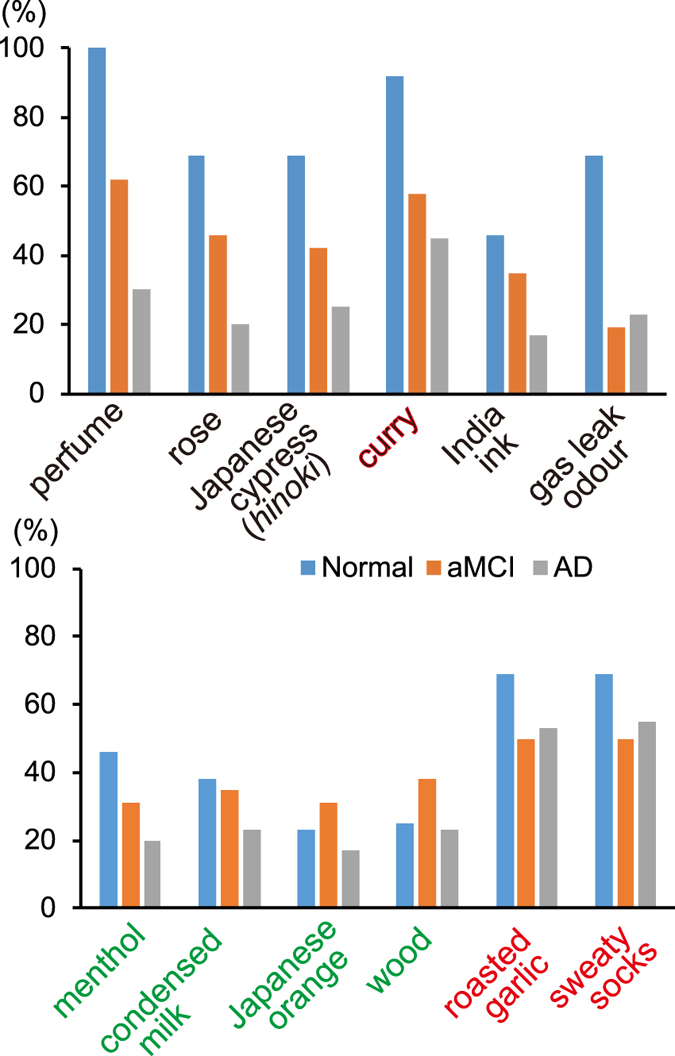
Accuracy of identification of each odorant by cognitive status. Odorants in the upper column became harder to accurately identify with declining cognitive status. *Green*, *black* and *red* odour names indicate Category I, Category II and Category III, respectively. (*See* Discussion).

The identification accuracy for each odorant in the Normal, aMCI and AD groups is shown in Fig. 2. Approximately half the odorants became harder to identify with disease progression. Interestingly, roasted garlic and sweaty socks were identifiable for many AD patients, while some other odorants were not identifiable even for normal aged subjects.

The Jonckheere-Terpstra test^13^ showed significant dementia progression-associated trends of declines in the identifiability of six odorants (Table 3): perfume, rose, Japanese cypress (*hinoki*), curry, India ink and gas leak odour. We defined an OSIT-J “*cognitive subset*” containing these six odorants and an OSIT-J “*non-congnitive subset*” containing the remaining six odorants: sweaty socks, roasted garlic, condensed milk, wood, Japanese orange and menthol.

**Table 3:** OSIT-J odorants and trends of declining identifiability with dementia progression by Jonckheere-Terpstra test.

Odorant	*Z* value	*p*
perfume	4.70	<0.001
rose	3.33	<0.001
Japanese cypress (*hinoki*)	2.97	0.003
curry	2.76	0.006
India ink	2.54	0.01
gas leak odour	2.07	0.04
menthol	1.93	0.053
condensed milk	1.37	0.17
Japanese orange	1.20	0.23
wood	1.20	0.23
roasted garlic	0.55	0.58
sweaty socks	0.40	0.69

The odour stick identification test for Japanese (OSIT-J) “*Cognitive subset*” consists of the six odorants that showed a significant trend of decline in identifiability with dementia progression.

**Figure 3:**
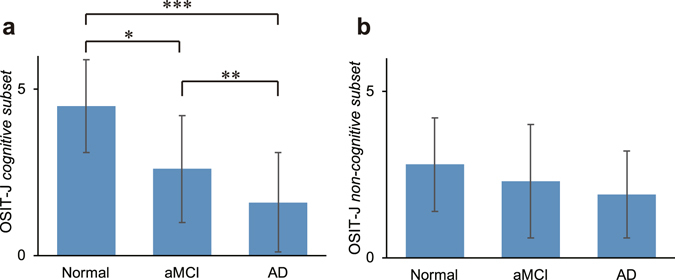
OSIT-J and cognitive status. (**a**) OSIT-J “*cognitive subset*” score (6 items: perfume, rose, Japanese cypress (*hinoki*), curry, India ink and gas leak odour) for each cognitive status. (**b**) OSIT-J “*non-cognitive subset*” score (6 items: sweaty socks, roasted garlic, condensed milk, wood, Japanese orange and menthol) for each cognitive status. The ability to identify the “*cognitive subset*” was a better indicator of cognitive status. Error bars are standard deviation. *, ** and *** denote significant (*p* < 0.05, *p* < 0.01 and *p* < 0.001, respectively) differences with Bonferroni correction. Abbreviations: OSIT-J, Odour Stick Identification Test for Japanese.

**Figure 4:**
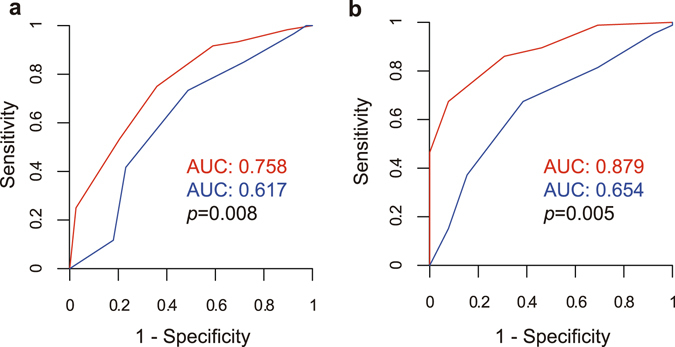
ROC curves of “*cognitive*” and “*non-cognitive*” OSIT-J subsets. *Red line* and *blue line* indicate the “*cognitive subset*” and “*non-cognitive subset*”, respectively. (**a**) ROC curves to discriminate AD vs (aMCI + Normal). The AUCs of “*cognitive subset*” and “*non-cognitive subset*” were 0.758 and 0.617, respectively. The difference was significant (*p* = 0.008). (**b**) ROC curves to discriminate (AD + aMCI) vs Normal. The AUCs of “*cognitive subset*” and “*non-cognitive subset*” were 0.879 and 0.654, respectively. The difference was significant (*p* = 0.005). Abbreviations: ROC, receiver operating characteristic; AUC, area under the curve; OSIT-J, Odour Stick Identification Test for Japanese; AD, Alzheimer’s disease; aMCI, amnestic mild cognitive impairment.

The ability to identify the “*cognitive subset*” odorants was a much better indicator of cognitive status than the ability to identify the “non-cognitive subset” odorants (Fig. 3). There were significant differences between the two receiver operating characteristic (ROC) curves for detecting AD (*i*.*e*. AD vs aMCI + Normal, *p* = 0.008) (Fig. 4a) and the two ROC curves for detecting cognitive decline (*i*.*e*. AD + aMCI vs Normal, *p* = 0.005) (Fig. 4b).

There were also significant differences in the identifiability of gas leak odour among the Normal (69% correct), aMCI (19%) and AD (23%) groups (Fig. 2), while curry, sweaty socks and roasted garlic were detectable for over 45% of AD patients.

## Discussion

The AD-associated olfactory decline affected each odorant differently. Three categories can be defined:

- Catetory I – odorants that were difficult for normal aged subjects to identify, including menthol, condensed milk, Japanese orange and wood;
- Category II – odorants that became harder to identify with cognitive decline, including perfume, rose, Japanese cypress (*hinoki*), curry, India ink and gas leak odour;
- Category III – odorants that even AD patients could identify, including roasted garlic, sweaty socks and (curry).

Curry became harder to identify with dementia progression, but it was still identifiable for 45% of AD patients. We therefore think that curry lies midway between Categories II and III (Fig. 2). This was consistent with another report that showed a significant difference between control and AD groups in accurately identifying India ink, rose, roasted garlic, wood and Japanese cypress (*hinoki*)^14^. Parkinson’s Disease (PD) patients also retained the ability to identify curry, sweaty socks, roasted garlic and perfume, showing a similar tendency to AD patients, with the exception of perfume^15^. This may reflect a common mechanism impacting olfaction in PD and AD.

The OSIT-J “*cognitive subset*” provided better disease biomarkers than the “*non-cognitive subset*” and would provide a more effective way to detect cognitive decline. Olfactory function tests restricted to cognitive decline-sensitive odorants would provide an inexpensive and simple clinical AD biomarker. We do not know why some odorants are identifiable to AD patients while others are not, but this might be associated with cholinergic modulation of olfaction^10^. Conspicuous odorants might not need cholinergic modulation that is damaged in AD.

It would be a great boon for AD patients if they could retain the ability to identify some odorants. Sweaty socks, roasted garlic and curry (Category III) were identified by 55, 53 and 45% of AD patients, respectively (Fig. 2). Olfaction affects taste mainly via retronasal olfaction pathways^16^, and strongly affects affected food enjoyment^17^. Harm to health may results from weight loss due to decreased appetite, or weight gain due to consumpting a sweet desert as a “gustatory” reward following an unexciting meal^18, 19^. Although retronasal and orthonasal olfaction are different in some ways^20^, they share a common basis. Our results suggest that fermented soybeans (*natto*, whose odorant, *isovaleric acid* is found in sweaty socks^12^), roasted garlic and curry may benefit AD patients in terms of taste enjoyment.

We need to reconsider the choice of odorant used to signal gas leaks. Combustible gases such as methane, ethane and propane have no odour. Gas odorants such as tert-butyl mercaptan and dimethyl sulfide have been added to household gas to make leaks detectable. We found that over 70% of aMCI and AD patients could not identify these odours of gas, which was consistent with previous studies^14, 21^. It should be noted that the ability to identify the gas leak odorant decreased as early as the aMCI level. On the IADL, 75% of such patients’ families reported that they prepare meals alone (data not shown), meaning that they cook without the ability to detect gas leaks. If aMCI patients are unaware of their hyposmia, they might ignore a gas detector alarm. Failure to detect gas leaks is among the most common impairments in patients with olfactory disabilities^21^. Using odours as gas leak signals was reported to be clearly unreliable for old people more than 50 years ago^22^.

This study confirmed that olfactory function mainly reflected recall ability. It has been proposed that the earliest pathologic changes of AD occur in the olfactory pathway^23^, which contains the anterior olfactory nucleus, prepiriform cortex, entorhinal cortex, amygdaloidal areas, hippocampal areas and orbitofrontal cortex^24^. It has also been revealed that the medial temporal lobe including the hippocampus and entorhinal cortex plays an important role in memory^25^.

This study has several limitations. Several OSIT-J odorants are familiar to Japanese people but may not be universal^12^. Although cognitive status was diagnosed by experienced geriatricians with the aids of medical imaging, some patients might have been miscategorized due to lack of amyloid imaging^26, 27^. We did not confirm whether aMCI patients progressed to AD. A longitudinal study may elucidate the progression to AD and whether olfactory dysfunction precedes cognitive decline. We recruited a limited number of normal subjects because we recruited from patients who were admitted for cognitive impairment evaluation. Finally, we cannot rule out the possibility of inconsistent odorant concentrations, although the manufacturer of the OSIT-J claims that young subjects obtain high scores.

## Conclusion

- A subset of odorants became less detectable with cognitive decline and can indicate cognitive status.
- Some smells could be detected even by AD patients.
- The ability to identify gas leak odorants decreases as early as the aMCI stage.

## Methods

### Patients

We enrolled 100 consecutive patients (30 men, 70 women, 65 to 93 years old) who were admitted to the Department of Geriatric Medicine, The University of Tokyo Hospital, Tokyo, Japan, for evaluation of cognitive impairment between January 2010 and March 2015. All patients were Japanese. We excluded patients who smoked, drank heavily, had undergone chemotherapy or had a nasal disease diagnosed by an otorhinolaryngologist or an interview. All participants were confirmed to be nasal disease-free using magnetic resonance imaging (MRI) or X-ray computed tomography (CT). We also excluded patients with non-AD dementia such as dementia with Lewy bodies (DLB), because the nature of AD-associated olfactory impairment may be different from that of non-AD. DLB patients usually show more severe anosmia than AD patients^28^.

This study was conducted in accordance with Ethical Guidelines for Medical and Health Research Involving Human Subjects in Japan and conformed to the Helsinki Declaration. The study protocol was approved by the institutional review board of the School of Medicine, the University of Tokyo. We provided patients and their families with detailed information and all participants provided written informed consent.

All patients were diagnosed by experienced geriatricians using DSM-IV criteria for AD, and Petersen’s operational criteria^29^, modified by Winblad *et al*.^30^ and Petersen *et al*.^31^ for aMCI. We made precise diagnoses using MRI or X-ray CT. [^123^I] N-isopropyl-p-iodoamphetamine (IMP) brain perfusion single photon emission computed tomography (SPECT) was also used. We excluded dementia with Lewy body with the aid of [^123^I] metaiodobenzylguanidine (MIBG).

### Cognitive and lifestyle scales

Cognitive levels of all patients were assessed using MMSE^32^. Their family members underwent IADL and the Barthel Index. We used the same 8-item IADL for both males and females.

### Olfactory testing

Olfactory function was examined using an OSIT-J test (Daiichi Yakuhin Sangyo, Tokyo, Japan) A significant correlation coefficient (0.701, *p* < 0.01) between the OSIT-J identification rate and the Cross-Cultural Smell Identification Test (CC-SIT) has been reported. The average estimated odorant familiarity score was significantly higher for the OSIT-J than the CC-SIT^12^. This test included 13 odorants consisting of essential oils, pure chemicals, mixed odorants and one negative control. Participants chose 1 of 6 possible answers: 3 incorrect odour names, 1 correct name, “unknown” or “no smell detected”. Participants were directed to avoid eating for 30 minutes before the examination.

### Statistical analyses

All analyses were performed using SAS (SAS Institute Inc., Cary, NC, USA). The data were analyzed in three groups: Normal, aMCI and AD groups.

Multiple regression analysis was performed to assess the relationship between OSIT-J and MMSE scores with age and sex as covariates. Hypertension and diabetes were also included as covariates to exclude the known effect of their relationship on olfactory function^33^. Olfaction also diminishes with age^34^.

We performed the Jonckheere-Terpstra Test^13^ to check whether the ability to accurately identify each odorant declined with dementia progression (*i*.*e*. Normal → aMCI → AD). We defined a subset of OSIT-J odorants that became harder to detect with cognitive decline as the “*cognitive subset*”, and the remaining subset as the “*non-cognitive subset*”. We confirmed the utility of “*cognitive subset*” by comparing area under the curve (AUC) of ROC curves to predict the subjects’ status (Normal vs aMCI/AD, Normal/aMCI vs AD) with “*non-cognitive subset*.
